# AI-mediated participatory reading and relational well-being: a conceptual framework from contemporary China

**DOI:** 10.3389/fpubh.2026.1875633

**Published:** 2026-08-19

**Authors:** Bingnan Li

**Affiliations:** School of Journalism and Communication, Shaanxi Normal University, Xi'an, China

**Keywords:** AI-mediated participatory reading ecology, China, cultural participation, digital reading, public mental health, relational well-being, social connection

## Abstract

This article develops a conceptual model of how AI-mediated reading practices reshape the social and relational conditions through which reading may contribute to well-being in contemporary China. Focusing on new mass literature and arts, it examines reading not only as textual reception but also as a practice increasingly connected to algorithmic selection, generative response, editorial mediation, and reading-centered community interaction. Through a theory-synthesis and model-building conceptual analysis based on a purposive narrative review, the article proposes the “AI-mediated participatory reading ecology”: a bounded framework that traces a sequential relationship among reading-centered encounters, generative responses, mediating publishing, participatory interaction, and possible relational outcomes. The framework does not claim that AI-mediated reading directly improves mental health. Instead, it specifies the conditions under which reading-related participation may support relational well-being through recognition, perceived belonging, expression, and meaningful social connection. It formulates four analytically examinable propositions concerning textual anchoring, mediated visibility, interaction quality, and stratified participation. The Chinese case is significant because platformized cultural circulation, intelligent publishing, commercial infrastructures, and public cultural agendas converge with particular intensity. At the same time, AI-mediated reading may intensify content overload, homogenization, unequal visibility, attention capture, and the exclusion of less connected groups. The article’s contribution lies in clarifying how AI-mediated infrastructures reshape the psychological and relational conditions under which reading may support social connection and well-being, while converting a broad descriptive category into a bounded mechanism model with explicit conditions, limits, and an empirical research agenda.

## 1 Introduction: reading transformation, AI, and the reorganization of cultural participation

Social connection is increasingly recognized as a central dimension of well-being. Beyond formal interventions, everyday cultural practices such as reading, shared interpretation, and participation in reading-related communities may provide opportunities for meaning-making, recognition, and belonging. Yet, the conditions through which reading generates these relational experiences are being reorganized by artificial intelligence, recommendation systems, and platform infrastructures. Understanding how AI reshapes these processes is therefore important for examining how cultural participation may support, weaken, or stratify well-being. In that sense, this article engages public mental health only at the level of socially distributed conditions of connection, exclusion, and relational support, rather than at the level of clinical intervention or diagnosis.

Reading is increasingly embedded in digital infrastructures that do more than deliver texts to readers. Platforms recommend works, organize visibility, sort attention, collect feedback, and enable readers to comment, share, rewrite, and sometimes co-create around cultural content (1–6). Under these conditions, reading cannot be understood only as private textual reception. It must also be examined as a mediated cultural practice situated within platform systems, publishing arrangements, and communities of participation.

This article examines that transformation through the case of China’s new mass literature and arts. Its central question is not whether artificial intelligence improves mental health in any direct or clinical sense. Rather, it asks how AI-mediated reading environments reorganize the relationship among reading, cultural participation, intelligent publishing, and social connection, and under what conditions these processes may support relational well-being. The article’s argument is that AI does not simply add efficiency to existing reading practices. It reconfigures the mediating conditions of reading by linking algorithmic selection, generative assistance, editorial infrastructure, and community interaction. In this sense, participatory reading is best understood not as unrestricted platform activity but as a bounded ecology in which reading remains the organizing practice while becoming increasingly entangled with circulation, evaluation, and limited forms of co-production.

Existing scholarly discussions of China’s new mass literature and arts have provided important accounts of media transformation, popular participation, and technological empowerment (7–9). Research on intelligent and platformized publishing has separately examined changes in editorial processes, distribution, and publishing infrastructure (10–14). Yet, these discussions have been less successful in explaining how reading itself changes when textual reception becomes increasingly bound up with platform visibility, recommendation, interaction, and intelligent publishing. Adjacent debates in reading studies, platform studies, and publishing research therefore tend to move along separate tracks. As a result, the relationships among reading, mediation, and social participation remain under-theorized.

### 1.1 Conceptual approach and analytical procedure

This article adopts a theory-synthesis and model-building conceptual design based on a purposive narrative review (15). It is neither a systematic review nor an empirical case study, and it does not offer survey evidence, platform data, psychological measurement, or causal testing. The literature corpus was assembled purposively and iteratively across four domains: reading and digital social reading; participatory culture and platformization; publishing and intelligent publishing; and relational well-being and public mental health. Search concepts combined terms such as reading, digital reading, social reading, participatory culture, platformization, intelligent publishing, generative AI, relational well-being, social connectedness, and their Chinese-language equivalents. Sources were identified through problem-oriented keyword searching and backward and forward citation tracing. Selection was based on direct conceptual relevance to definitions, mechanisms, boundary conditions, or the Chinese institutional context rather than on exhaustive coverage. Priority was given to peer-reviewed articles, scholarly books, and authoritative institutional reports in Chinese and English. Journalistic, trade, institutional, or platform materials, where retained, are used only to document the circulation of terms or the stated configuration of particular systems, not to establish the article’s central theoretical claims or their social effects.

The analysis proceeded through four conceptual operations. First, concept differentiation separated reading, cultural participation, social connectedness, relational well-being, and public mental health to prevent these terms from being treated as interchangeable. Second, cross-field synthesis identified recurring mechanisms across the four literatures: textual encounters, generative responses, publishing mediation, platformed participation, and relational outcomes. Third, boundary testing examined conditions under which the proposed relationships may weaken or reverse, especially unequal access, algorithmic narrowing, content overload, extractive engagement design, and asymmetrical moderation. Fourth, model construction organized these mechanisms into a sequential framework and formulated propositions to guide empirical examination. The Chinese case functions as a concept-generating context, while international scholarship is used to assess which mechanisms are portable and which remain institutionally specific.

Because the synthesis is problem-driven rather than exhaustive, the article does not claim comprehensive coverage of every relevant body of literature. Its analytical rigor instead rests on making the selection logic, conceptual operations, inferential steps, and boundary conditions explicit. The aim is not to prove therapeutic outcomes but to clarify a conceptual relationship and specify when AI-mediated participatory reading may or may not support relational well-being.

The remainder of the article is organized into seven sections. Section 2 differentiates the focal concepts. Section 3 situates the argument in China’s new mass literature and arts. Section 4 distinguishes three forms of AI mediation and explains intelligent publishing as reading infrastructure. Section 5 develops the bounded model and its propositions. Section 6 analyzes opportunities, risks, inequalities, and governance conditions. Section 7 discusses scope, limits, and international relevance. Section 8 concludes with an empirical research agenda.

## 2 Reading, cultural participation, and relational well-being

Several concepts in this article require careful differentiation. “Reading” here refers to a practice centered on engagement with narrative, textual, or multimodal cultural works, even when that engagement extends into commenting, annotation, sharing, interpretive exchange, and limited co-creative response. It does not refer to all forms of platform participation. “Relational well-being” refers to the social and cultural conditions through which reading may support recognition, belonging, expression, and meaningful connection. It is related to, but not identical to, mental health. “Public mental health” is used here only in a broad contextual sense to indicate that mental well-being is shaped by social and cultural environments, not to claim that AI-mediated reading functions as a clinical intervention or as a demonstrable population-level health outcome. These distinctions are essential if participatory reading is to remain analytically precise rather than becoming a general label for digital interaction.

The relationship among these concepts is sequential rather than synonymous. Reading is the focal cultural practice. Cultural participation describes the extension of that practice into shared interpretation, circulation, and response. Social connectedness is one possible proximate mechanism generated through such participation. Relational well-being is a broader evaluative condition concerning whether people experience recognition, reciprocity, belonging, and an ability to act within supportive relationships (16, 17). Public mental health remains the widest contextual horizon and cannot be inferred directly from any one reading practice. This sequence prevents the article from moving uncritically from participation to connection, from connection to well-being, or from well-being to population-level mental health.

Reading has long exceeded the narrow boundaries of literacy acquisition or information extraction. It can function as a leisure practice, a mode of self-formation, a means of entering shared imaginative worlds, and a vehicle of cultural participation. In this broader sense, reading matters not only because it transmits knowledge, but because it can organize attention, foster interpretation, and create opportunities for identification, reflection, and dialog (1–4, 18–20). This wider understanding is especially important in digital environments where reading increasingly unfolds across interfaces, communities, and feedback systems rather than in isolation.

Scholarships on the arts, health, and well-being provide one reason for taking this broader view seriously. Research on shared reading, bibliotherapy, and library-based mental health support has shown that reading may matter for emotional coping, companionship, and meaning-making under certain institutional conditions (21–26). Yet, these findings should not be generalized too quickly. They do not establish that every reading practice improves well-being, nor do they justify direct claims about population-level mental health effects from platformized or AI-mediated reading. Their relevance to the present article is more modest: they show that reading can matter socially and affectively when embedded in supportive settings, and they open a legitimate pathway for asking how digitally mediated reading might reshape those settings.

The concept of relational well-being is useful precisely because it avoids collapsing these issues into therapeutic or clinical language too early. It highlights how reading may support expression, recognition, belonging, and meaningful connection through cultural participation. A reader may feel less isolated not because a platform produces mental health as an outcome, but because reading-related interaction provides companionship, interpretive exchange, or a sense of being recognized within a cultural public. Conversely, the absence of such conditions may leave technologically enabled participation socially thin or even exhausting.

This perspective also helps preserve what remains distinctive about reading. The point is not to redefine reading so broadly that it becomes synonymous with all online activity. Rather, the point is to ask what happens when reading-centered engagement extends into commentary, circulation, and visible response without losing its anchoring in works, narratives, and interpretive practices. Reading remains analytically central because it organizes the encounter with texts and cultural forms, even as that encounter now generates social traces, algorithmic signals, and sometimes derivative expression. Research on digital social reading likewise shows that online reviewing, annotation, discussion, and fan-based writing remain identifiable as reading-related practices when they continue to revolve around shared encounters with texts and their interpretation (6).

From this perspective, the article makes a deliberately bound claim. AI-mediated participatory reading may matter for relational well-being not because it directly treats psychological distress, but because it may reorganize the conditions under which people encounter texts, participate in communities, express themselves, and receive recognition. Whether those conditions become supportive, extractive, or exclusionary is both an empirical and normative question rather than one that can be resolved *a priori*. This conceptual starting point also clarifies why the Chinese case matters: it makes these transformations especially visible at the intersection of platformization, intelligent publishing, and public cultural participation.

## 3 China’s new mass literature and arts as a reading context

China’s new mass literature and arts should be understood not simply as a new label for online popular culture, but as a historically specific formation emerging at the intersection of platformized cultural production, digitally expanded participation, and renewed discussion of people-oriented cultural expression (7–9). In this context, the term refers to a broad field of literary and artistic practices that includes online literature, micro-dramas, short-form video storytelling, audio works, social-media micro-writing, fan-based rewriting, and other forms of vernacular cultural production that circulate through digital platforms and increasingly interact with AI-assisted tools. What makes this field analytically significant is not only the diversity of its media forms but also the way it reorganizes the relationship among readers, creators, intermediaries, and systems of visibility. From a well-being perspective, this transformation matters because cultural participation is increasingly mediated by infrastructures that shape not only the circulation and discoverability of cultural content but also how readers experience recognition, belonging, and connection. The literary-magazine discussion is used solely to trace the public circulation of the term (27); the definition and analytical claims developed here rest on scholarly research rather than on media sources.

The Chinese context matters here for more than illustrative reasons. First, platformization has become a major condition of cultural circulation, meaning that reading, evaluation, recommendation, and participation are increasingly organized through integrated commercial and communicative infrastructures rather than through distinct stages of production, distribution, and reception (5, 12, 13). Second, publishing in China is being reshaped not only by digitization but also by the expansion of intelligent publishing, data-driven distribution, and platform-based cultural mediation, which increasingly connect editorial processes with metrics, recommendations, and user responses (10–14). Third, the field is shaped by a distinctive combination of public cultural discourse, commercial platform competition, and institutional concern with the social reach, accessibility, participation, and inclusiveness of literary and artistic life (7–9). For these reasons, the Chinese case provides a particularly illuminating site for examining how reading is moving beyond private reception toward forms of visible, interactive, and platform-conditioned participation.

The value of this context lies not merely in its scale, novelty, or technological speed. It lies in the fact that multiple transformations often analyzed separately in other contexts are unfolding together here: reading is becoming more visible, publishing more infrastructural, participation more platform-dependent, and cultural legitimacy more closely tied to systems of circulation and discoverability. This conjunction makes China especially useful for conceptual analysis because it reveals how reading can remain text-centered while becoming inseparable from platform governance, mediated visibility, and the social conditions through which participation and belonging are experienced.

What is analytically distinctive is therefore not that recommendation, co-creation, or platform participation occur only in China. These mechanisms are internationally recognizable. The more context-specific feature is their articulation with the discourse of new mass literature and arts, the continuing institutional role of publishing and cultural policy, and the hybrid relationship between commercial platform infrastructures and public cultural agendas. The Chinese case should thus be treated as a particular configuration of portable mechanisms and locally specific institutions, rather than a wholly exceptional system or a simply illustrative national example.

Contemporary Chinese publishing and reading systems make these layers visible in different ways, but they are not used here as empirical tests of the model. Publisher-side systems may combine content processing, editorial assistance, rights coordination, knowledge resources, and workflow management; literary platforms may aggregate reader feedback and performance metrics; and social-reading interfaces may organize reader ratings, reviews, annotations, and discoverability. These configurations illustrate the distinction between publisher-side intelligent mediation, creator-platform-reader feedback, and reading-centered interaction. They do not demonstrate that any particular system improves reading quality, cultural inclusion, social connection, or well-being. In keeping with journal guidance on nonacademic and commercial sources, platform materials are minimized and used only for the limited functions described earlier; the article’s mechanism claims rest on the scholarly literature cited throughout this section and Sections 4–7.

This transformation also complicates the conventional division of cultural labor. Readers do not simply receive finished works; they comment, annotate, reward, circulate, adapt, and sometimes feed ideas back into ongoing production (4, 18, 19). At the same time, creators and publishers increasingly respond to platform signals, community feedback, and visibility metrics. The result is not the disappearance of mediation, but its redistribution across platforms, editorial systems, and user-facing interfaces (5, 11–14, 28). Once these changing relations are taken seriously, publishing can no longer be treated as a secondary after-effect of creation; it must be analyzed as one of the central infrastructures through which reading is organized and made visible.

At the same time, this shift should not be overstated as pure democratization. Expanded participation does not eliminate hierarchy; it reorganizes it. Platform visibility, traffic logics, editorial filtering, and algorithmic recommendation continue to shape whose stories circulate, whose voices are amplified, and what forms of expression gain legitimacy. Research on platformized cultural industries further shows that creativity and participation are inseparable from transformations in labor, governance, infrastructure, and citizenship (29). The significance of new mass literature and arts therefore lies not in proving that all readers have become creators, but in showing that the boundaries between reading, response, circulation, and cultural production have become more permeable under specific institutional and technological conditions. It is precisely this permeability, rather than a total collapse of distinctions, that makes the Chinese case especially valuable for conceptual analysis.

## 4 AI-mediated reading infrastructure: intelligent publishing, recommendation, and generative assistance

In this article, “AI-mediated” does not refer to every digital process involved in online culture. It refers more specifically to three forms of mediation that are especially relevant to participatory reading: algorithmic selection, through which platforms rank and distribute cultural content; generative assistance, through which users and creators employ AI tools for drafting, rewriting, summarizing, transforming, or extending works; and intelligent publishing mediation, through which editorial, classificatory, and recommendation systems organize reading pathways and content visibility (5, 14, 30–32). These forms of mediation overlap in practice, but they are not analytically identical. Distinguishing them helps prevent “AI” from operating as an undifferentiated prestige term and allows the analysis to specify which mechanisms are most relevant to reading, participation, and cultural evaluation.

This distinction matters because publishing should not be treated as a downstream technical step that merely follows creation. Publishing is one of the key mediating arrangements through which works become selectable, legible, discoverable, and publicly consequential (5, 28, 33, 34). In digital environments, this mediating function is increasingly distributed across metadata systems, platform interfaces, editorial procedures, recommendation logics, and metrics of engagement (10–14). These processes also shape readers’ cognitive and emotional experience by influencing accessibility, perceived autonomy, discoverability, and the effort required to engage with cultural works. Intelligent publishing therefore matters not only because it may improve efficiency, but because it helps organize the conditions under which reading takes place.

Seen this way, intelligent publishing performs at least four interrelated functions in AI-mediated reading environments. First, it supports production by lowering certain thresholds of drafting, revising, formatting, multimodal transformation, and accessibility. Second, it mediates discovery by shaping how works are classified, surfaced, and sequenced for readers. Third, it participates in evaluation by linking editorial judgment, ranking systems, and visible feedback signals. Fourth, it contributes to governance by structuring attribution, disclosure, copyright coordination, and the balance between openness and quality control (5, 10–14, 28, 32). These functions should be understood as potential infrastructural operations rather than as demonstrated effects of any particular platform.

This is why intelligent publishing cannot be reduced to a neutral efficiency tool. The same systems that widen access may also narrow visibility. The same recommendation pathways that help readers find relevant works may also reinforce genre routines, commercial preference, or short-cycle engagement. Likewise, generative assistance may support expression for new participants while also amplifying templated language, stylistic convergence, or dependence on machine-supported production (2, 3, 5, 30–32, 35). The question, then, is not whether intelligent publishing is good or bad in the abstract, but how its mediating role shapes the reading ecology within which participation becomes possible.

For the purposes of this article, platform examples should therefore be understood as illustrative rather than evidential. The point is not to prove that any specific AI-enabled system causes better reading outcomes, but to clarify how publishing infrastructures mediate the relationships among textual encounter, circulation, participation, and cultural evaluation. This mediation is precisely what makes reading both an interpretive and an infrastructural practice in the age of AI. On that basis, the next step is to specify how these mediated relations can be conceptualized without turning the framework into an all-purpose description of digital culture.

## 5 Findings: a bounded model of AI-mediated participatory reading

The concept of AI-mediated participatory reading ecology is most useful when treated not as a total description of digital culture, but as a bounded model for analyzing how reading-centered participation is reorganized under AI-mediated conditions. Its purpose is not to absorb every aspect of online interaction into a single framework. Rather, it identifies four analytically distinct but interrelated dimensions through which reading is transformed after an initial textual encounter: generative reading response, mediating publishing, reading-centered participation, and relational well-being. These dimensions should be understood as levels of analysis rather than a simple list of features.

Generative reading response refers to the extension of reading into forms of response that may feed back into cultural production. It includes commenting, annotating, sharing, rewriting, prompted continuation, and other practices through which readers not only interpret texts but also leave traces that may influence circulation, visibility, and in some cases subsequent creation (4, 6, 18, 19). However, generative reading response does not mean that all reader activity becomes authorship. Its significance lies in showing that reading increasingly produces actionable signals and culturally meaningful responses within platformized environments.

Mediating publishing refers to the infrastructural and editorial processes that organize how works are selected, formatted, classified, recommended, and evaluated (5, 11–14, 28, 33, 34). This dimension is essential because participatory reading does not unfold in an unstructured space. Intelligent publishing systems, editorial judgment, metadata organization, recommendation pathways, and platform rules all shape what becomes readable, discoverable, and discussable. Treating publishing as mediation helps distinguish the framework from more general theories of digital participation by foregrounding the institutions and infrastructures that structure reading.

Reading-centered participation refers to the social dimension through which reading becomes visible, collective, and circulatory (4, 5, 18). Readers participate not only by consuming texts but also by entering communities of interpretation, affect, and exchange. Yet participation here should still be delimited: the framework concerns reading-centered participation, not every act of online belonging or user activity. The analytical question is not whether platforms enable interaction in general, but how reading-related interaction becomes a site of expression, recognition, negotiation, and contestation.

Relational well-being names the most cautious and conditional dimension of the model. It does not describe an automatic outcome, nor does it imply clinical benefit. Rather, it identifies the social and cultural conditions under which reading-centered participation may support expressive opportunity, belonging, recognition, and meaningful connection (20–26). This dimension is placed last deliberately: well-being is not assumed at the outset, but emerges only if the preceding mediations operate in ways that support inclusion, quality, and sustainable interaction rather than metric-driven attention alone.

Taken together, the four dimensions form an explanatory sequence rather than a descriptive taxonomy. Reading extends into generative response; publishing mediates the conditions of circulation; participation gives those responses social form; and relational well-being becomes one possible, but never guaranteed, consequence. The model therefore gains analytical value not by claiming total novelty, but by specifying the mediated pathways through which reading may become participatory and conditionally consequential in the age of AI. Once those pathways are specified, the central issue is no longer whether AI creates participation in the abstract, but under what conditions the resulting ecology widens expression, deepens exclusion, or does both at once.

### 5.1 Conceptual propositions

To move beyond a descriptive taxonomy, the model generates four analytically examinable propositions.

#### 5.1.1 Proposition 1: reading anchoring

AI-mediated activity should be treated as participatory reading only when commenting, sharing, rewriting, or co-creation remains anchored in an identifiable encounter with a text, narrative, or multimodal cultural work. As sustained textual and interpretive anchoring weakens, the explanatory fit of the framework declines, and the activity is better understood as general platform participation.

#### 5.1.2 Proposition 2: mediated conversion

Generative reader responses do not become culturally consequential through expression alone. Their influence on circulation and participation depends on publishing and platform mediation, which classifies, evaluates, recommends, and makes those responses visible. Similar reader practices may therefore produce different participatory outcomes under different editorial and algorithmic regimes.

#### 5.1.3 Proposition 3: relational quality

Reading-centered participation is more likely to support relational well-being when interaction is reciprocal, sustained, recognitive, and interpretively substantive. The relationship is likely to weaken or reverse when interaction is episodic, competitive, hostile, or optimized primarily for short-cycle engagement metrics.

#### 5.1.4 Proposition 4: stratified participation

Inequalities in access, AI capability, language resources, moderation, and visibility condition every transition in the model. Where these inequalities are pronounced, expanded participation may coexist with reduced cultural inclusion and unevenly distributed relational benefits.

These propositions do not claim universal causal laws. They specify relationships that can be examined through comparative platform studies, interviews, digital ethnography, content analysis, and reader-centered research. Relevant indicators may include the degree of textual anchoring, pathways of editorial and algorithmic visibility, reciprocity and durability of interaction, and the distribution of access and recognition across social groups.

**Figure 1:**
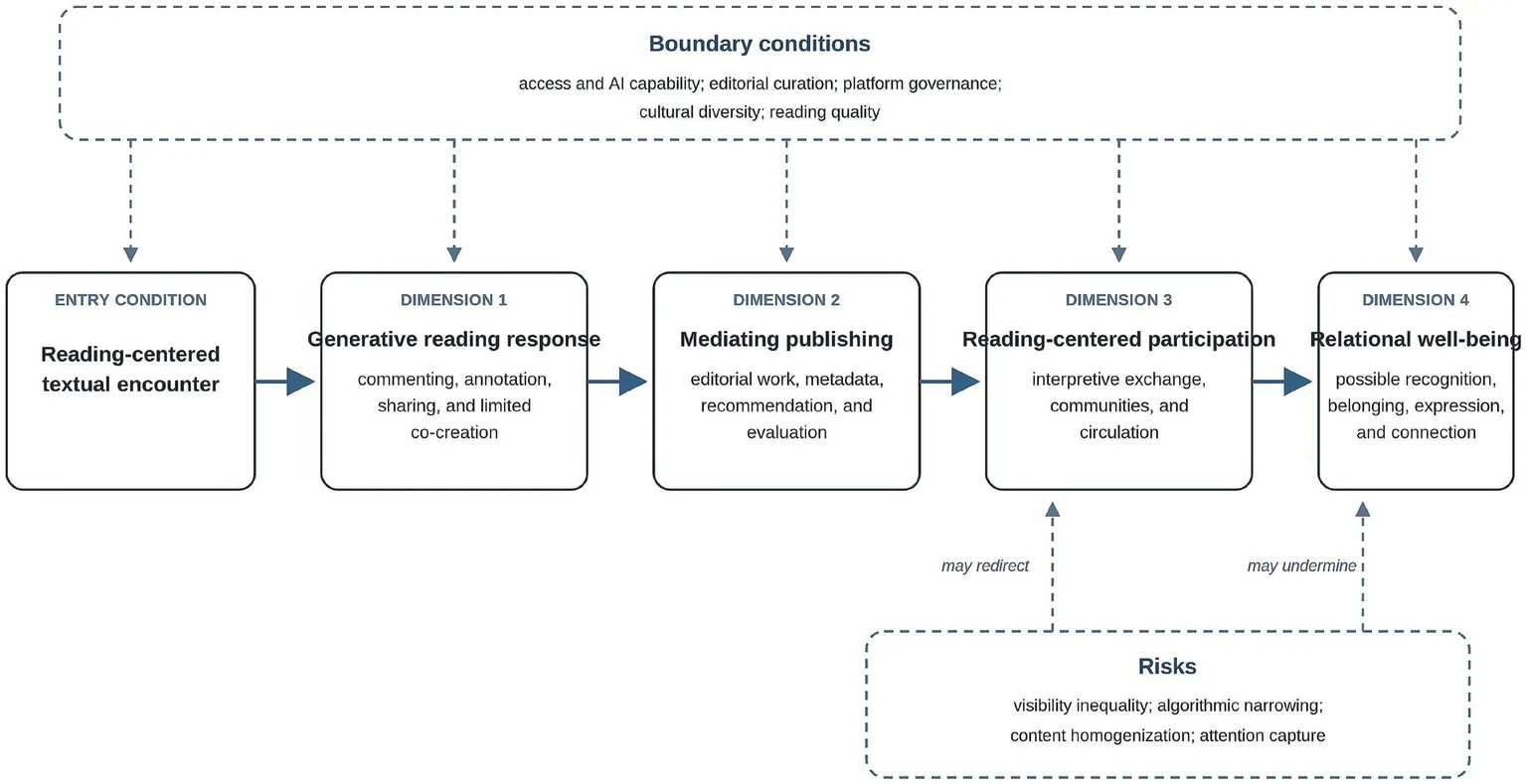
Bounded mechanism of AI-mediated participatory reading ecology. Solid arrows indicate the proposed sequential pathway. Dashed arrows indicate boundary conditions and risks that may condition, redirect, or undermine the pathway. Source: Author’s conceptualization.

The mechanism contains five visible stages but only four analytical dimensions. *Reading-centered textual encounter* is the entry condition of the model; it is followed by the four dimensions developed above: generative reading response, mediating publishing, reading-centered participation, and relational well-being. Figure 1 uses shortened box labels for visual clarity.

### 5.2 Opportunities, risks, and governance conditions

The opportunities associated with AI-mediated participatory reading should be framed in conditional rather than celebratory terms. Creative access may widen when readers and non-professional creators can use AI-assisted tools to draft, revise, narrate, or transform cultural expression into more publicly legible forms. Reading communities may deepen when interaction produces recognition, sustained exchange, and interpretive companionship rather than fleeting response. Publishing may recover an important public role when mediation is oriented toward diversity, quality, and accessibility rather than visibility metrics alone. None of these outcomes follows automatically from technological adoption. Each depends on editorial design, platform norms, access conditions, and the broader value orientation of cultural infrastructures.

One central counter mechanism is the abundance paradox. Lower thresholds for production can widen expressive opportunity while simultaneously producing more repetitive, weakly attributed, or low-quality material. As the volume of AI-assisted content grows, readers face greater filtering pressure and selection fatigue, while platforms and publishers gain additional power as curators of discoverability. Generative systems may also reproduce dominant styles and high-frequency patterns, so a larger supply of content does not necessarily yield greater cultural diversity (30–32). Content overabundance therefore makes editorial mediation more necessary at the same time that it increases the risks attached to concentrated gatekeeping.

A second counter mechanism concerns the capture of participation as labor and data. Reviews, recommendations, annotations, community moderation, emotional performances, and promotional circulation can create value for reading communities, but they also supply platforms with unpaid cultural labor, behavioral data, and attention. Platformization thus reorganizes creativity and participation together with labor and governance (29, 35). Relational value may coexist with affective extraction: users can experience belonging even as they are pressured to remain visible, continuously responsive, and metrically productive. This tension means that well-being cannot be assessed only through the presence of interaction; it must also consider who captures the value generated by that interaction and what forms of burden participation imposes.

This conditional framing is especially important because the same ecology may intensify exclusion while appearing to expand participation. Empirical research on digital assistants shows that inequalities in AI access and use track income and education, and that unequal access is also associated with differences in users’ understanding of algorithmic bias, misjudgment, and surveillance (36). Access to AI tools is therefore unevenly distributed not only across region, age, education, income, language resources, and digital literacy, but also across levels of critical and practical capability. Even where baseline access exists, meaningful participation may remain stratified by access to paid tools, advanced features, time, training, and platform familiarity. As a result, the rhetoric of openness can conceal a layered digital divide: some users participate through visible and technically enhanced forms of expression, while others remain peripheral, less discoverable, or effectively excluded from high-value cultural circulation. Chinese evidence reinforces the relevance of this concern. Research using data from the China Family Panel Studies finds that digital divides significantly constrain the social participation of rural older adults, with differences in use and knowledge operating alongside basic access and with cultural capital and social trust implicated in the mechanism (37). Although this study is not specifically about reading platforms, it shows why access alone is an insufficient measure of inclusion in the Chinese context.

Minority and marginalized perspectives require more explicit consideration within this framework. Cultural inclusion should not be measured solely by the number of participants entering a platform, but by whether different linguistic, ethnic, regional, generational, and ideological experiences can appear without being flattened into dominant formats of visibility. Platform systems may broaden access while still narrowing acceptable styles of expression through recommendation bias, moderation asymmetries, commercial preference, or the privileging of high-engagement genres (5, 31, 35). Research on #BookTok likewise shows that platform-based reading communities can create opportunities for peer learning while reproducing dominant cultural norms and uneven visibility (38). In such cases, participation remains real but unequal, and recognition becomes selectively distributed.

A similar ambivalence applies to community formation. Reading-related communities can indeed foster companionship, identity affirmation, and shared interpretation. Internationally, #BookTok provides a documented example of a creator-led reading community in which knowledge about books, reading identities, selection practices, and critical literacy is shared through peer pedagogies (38). Yet, the same research cautions that no platform community guarantees quality or ethical standards, and that dominant or harmful cultural norms may also be reinforced. Such communities can additionally generate affective pressure, performative self-presentation, traffic anxiety, and continuous comparison. Under traffic-oriented conditions, interaction may reward immediacy over reflection, intensity over depth, and repetition over interpretive complexity. What appears as community may therefore oscillate between meaningful belonging and metricized participation. The key question is not whether reading communities exist, but what kinds of attention, temporality, and relational norms they cultivate.

International digital social-reading research also documents a different configuration built around shared annotation and text-attached discussion rather than influencer-led circulation. In these environments, comments, highlights, and interpretive exchanges remain visibly anchored to particular passages or works (6). This contrast is useful because it shows that reading-centered participation can be organized through multiple platform architectures, and that the depth of textual anchoring should be examined rather than inferred from the mere presence of interaction.

These inequalities also clarify why governance cannot be treated as a final add-on to an otherwise positive ecology. Questions of access, visibility, moderation, and recognition are not external constraints; they are part of the mechanism through which participatory reading becomes either enabling or exclusionary. A framework concerned with reading-related well-being must therefore ask not only whether participation expands, but which groups are able to participate on meaningful terms, which forms of reading are rewarded, and which voices remain structurally harder to discover or sustain.

For this reason, governance should be framed less as the regulation of isolated tools than as the shaping of a reading ecology. At the level of access, governance must consider affordability, digital literacy, accessibility design, and the unequal distribution of AI capabilities across age, region, education, and linguistic communities. At the level of mediation, it should address transparency in recommendations, editorial curation, discoverability for less-represented voices, and the prevention of purely traffic-driven visibility hierarchies. At the level of participation, it should consider copyright, attribution, moderation fairness, and community norms that protect slower, more reflective, and less extractive forms of interaction. At the level of well-being, governance should ask not simply how to maximize engagement but how to cultivate cultural environments in which expression, recognition, and high-quality reading experiences remain possible without intensifying exhaustion or exclusion.

Table 1 consolidates these conditions without treating them as a checklist detached from the argument. Its rows correspond to the sequence developed above: access conditions shape who can enter; publishing and platform mediation shape what becomes visible; participatory norms shape the quality of interaction; and only then can relational well-being be considered a possible outcome. The table therefore summarizes the model’s boundary conditions and governance implications rather than supplying evidence independent of the main text.

**Table 1:** Opportunities, risks, and governance conditions in AI-mediated participatory reading ecology.

Analytical dimension	Conditional contribution	Principal risk	Governance condition
Access and generative assistance	Lower thresholds for drafting, adaptation, accessibility, and vernacular expression	Unequal access to devices, paid models, advanced features, time, skills, and linguistic resources	Affordability support; accessibility design; digital and AI literacy; attention to regional, age, educational, and linguistic differences
Publishing and discoverability	Improved classification, editorial support, reading pathways, and access to diverse works	Algorithmic narrowing; content overabundance; commercial metrics displacing cultural value	Recommendation transparency; editorial curation; plural evaluation criteria; discoverability for less-represented voices
Reading-centered participation	Interpretation, peer learning, identity expression, and sustained community exchange	Shallow engagement; unpaid or affective labor; moderation asymmetry; traffic anxiety and polarization	Fair moderation; attribution and copyright rules; community norms; spaces for slower and reflective reading
Cultural inclusion	Greater visibility for local, minority, and everyday narratives	Dominant formats flattening linguistic, ethnic, regional, generational, or ideological difference	Inclusive design; minority-language support; visibility audits; participatory governance
Relational well-being	Recognition, belonging, expressive opportunity, and meaningful connection	Attentional exhaustion; social comparison; selective recognition; exclusion disguised as openness	Well-being-oriented evaluation; limits on extractive engagement design; reader-centered assessment

## 6 Discussion: theoretical contribution, scope, and international relevance

The contribution of AI-mediated participatory reading ecology does not rest on claiming that commenting, social reading, recommendation systems, or platformized cultural circulation are individually new. These phenomena have established intellectual lineages in reader-response theory, participatory culture, digital social reading, platform studies, and publishing research (4–6, 18, 19, 28, 29, 33, 34). The framework’s contribution lies instead in specifying their relationship around a reading-centered mechanism that is also health-relevant. It places publishing mediation between reader response and social participation, treats relational well-being as a conditional outcome rather than an inherent benefit, and identifies inequality and governance as mechanisms operating within rather than outside the ecology. In this respect, the model is a theory synthesis and reordering of existing insights rather than a claim to conceptual invention *ex nihilo*. Its relevance to a health-oriented readership lies in clarifying when AI-mediated reading environments may support or weaken the social conditions of recognition, belonging, and meaningful connection. For the purposes of this article, this article should be understood as a conceptual contribution to debates on social connection, exclusion, and everyday well-being under AI-mediated cultural conditions, rather than as a clinical or intervention study.

The distinction from established frameworks can be stated more precisely. From Jenkins, the model retains the importance of participation and co-creation but adds a *reading-anchoring requirement*, preventing participatory culture from becoming interchangeable with reading (4). From Benkler, it retains distributed contribution but adds the editorial and algorithmic processes through which contributions are converted into visibility and cultural consequence (18). From van Dijck and Poell, it adopts the insight that programmability, popularity, and connectivity structure participation, but narrows those logics to reading-centered pathways and restores publishing institutions as active mediators (5). From McKenzie, it retains the sociology of texts and the importance of mediation while extending that concern to generative systems, recommendation infrastructures, platform metrics, and conditional relational outcomes (28). The proposed framework is therefore not a replacement for these theories; it is a mechanism-level synthesis designed for the specific problem of AI-mediated reading.

The framework also has clear boundary conditions. It is most persuasive where reading remains the organizing practice, where participation can be traced to engagement with identifiable works, where publishing or platform mediation shapes visibility, and where interaction leaves durable traces in circulation or community formation. It is less useful for fleeting reactions, generic social networking, or content consumption that involves little sustained textual encounter or interpretive exchange. The distinction is not between solitary and social reading, since both may be meaningful, but between reading-centered practices and platform activities whose connection to reading is merely nominal.

A second boundary concerns the relation between relational well-being and public mental health. The model proposes a plausible pathway from reading-centered encounter to participation, recognition, belonging, and meaningful connection. It does not establish a direct transition from these experiences to clinical improvement or population-level mental health effects. Relational well-being is itself dynamic and situated: the same community may provide recognition to some participants while exposing others to comparison, hostility, exclusion, or fatigue (16, 17). The model should therefore be used to generate questions about conditions and mechanisms rather than for inferring therapeutic effectiveness.

The Chinese case combines mechanisms that are internationally portable with institutions that are more context-specific. Algorithmic recommendation, generative assistance, visibility metrics, digital social reading, and platform labor appear across national settings. More specific to the Chinese configuration are the contemporary discourse of new mass literature and arts, the continuing institutional role of publishing organizations and cultural policy, and the articulation of commercial platform systems with public cultural agendas. The international relevance of the case lies not in its representativeness but in its analytical contrast: it helps distinguish general platform mechanisms from the institutional arrangements through which those mechanisms acquire cultural meanings and governance priorities.

The framework is also open to revision. Its explanatory value would be weakened if empirical research found that the relevant outcomes were driven mainly by generic platform sociability rather than reading-centered engagement, that editorial and algorithmic mediation played little role in converting reader response into visibility, or that interaction quality and access inequality did not meaningfully condition relational outcomes. Stating these possibilities is important because a conceptual model should not be protected from empirical challenge by definitions broad enough to absorb every result.

Future research can operationalize the model at several levels. At the practice level, studies can examine the depth and continuity of textual engagement before and after commenting, remixing, or co-creation. At the infrastructural level, platform audits and publishing research can trace how metadata, ranking, recommendation, and editorial interventions shape discoverability. At the relational level, interviews and digital ethnography can investigate reciprocity, recognition, belonging, pressure, and fatigue. At the distributional level, comparative studies can test how age, region, education, language, income, disability, and access to paid AI capabilities affect participation and visibility. Cross-national comparisons would be especially valuable for separating platform-wide mechanisms from institutional features specific to the Chinese context.

## 7 Conclusion

This article has argued that the transformation of reading in contemporary China is best understood as a reorganization of mediation rather than simply a change of format or the addition of AI tools. The proposed framework makes three contributions. First, it preserves the distinctiveness of reading by requiring textual and interpretive anchoring. Second, it identifies publishing and platform mediation as the mechanism through which reader responses become visible and culturally consequential. Third, it treats relational well-being as a conditional and unequally distributed possibility rather than as an automatic mental health benefit. For health-oriented scholarship, the central implication is that AI matters not only because it changes access to texts but because it reorganizes the social and psychological conditions through which reading may support connection, belonging, and everyday well-being.

The framework is conceptual rather than causal. Its purpose is to identify mechanisms, propositions, and boundary conditions that can be tested, revised, or rejected through empirical research. China’s new mass literature and arts provide a valuable case study in this regard because they bring platformized circulation, intelligent publishing, commercial infrastructures, and public cultural agendas into a particularly dense configuration. The broader implication is that the future of reading in the age of AI will depend less on technological novelty than on how mediation, discoverability, participation, labor, inclusion, and cultural responsibility are organized. A reading ecology should therefore be evaluated not by the quantity of generated content or engagement alone but by the quality of textual encounter, the fairness of visibility, and the relational conditions it sustains.
